# Identifying clinical and biochemical phenotypes in acute respiratory distress syndrome secondary to coronavirus disease-2019

**DOI:** 10.1016/j.eclinm.2021.100829

**Published:** 2021-04-15

**Authors:** Sylvia Ranjeva, Riccardo Pinciroli, Evan Hodell, Ariel Mueller, C. Corey Hardin, B. Taylor Thompson, Lorenzo Berra

**Affiliations:** a Department of Anesthesia, Critical Care and Pain Medicine, Massachusetts General Hospital, 55 Fruit Street, Boston MA 02114, USA; b Pulmonary Critical Care Division, Department of Medicine, Massachusetts General Hospital, 55 Fruit Street, Boston MA 02114, USA

**Keywords:** ARDS, COVID-19, Phenotypes, Statistical inference

## Abstract

**Background:**

Acute respiratory distress syndrome (ARDS) secondary to coronavirus disease-2019 (COVID-19) is characterized by substantial heterogeneity in clinical, biochemical, and physiological characteristics. However, the pathophysiology of severe COVID-19 infection is poorly understood. Previous studies established clinical and biological phenotypes among classical ARDS cohorts, with important therapeutic implications. The phenotypic profile of COVID-19 associated ARDS remains unknown.

**Methods:**

We used latent class modeling via a multivariate mixture model to identify phenotypes from clinical and biochemical data collected from 263 patients admitted to Massachusetts General Hospital intensive care unit with COVID-19-associated ARDS between March 13 and August 2, 2020.

**Findings:**

We identified two distinct phenotypes of COVID-19-associated ARDS, with substantial differences in biochemical profiles despite minimal differences in respiratory dynamics. The minority phenotype (class 2, *n* = 70, 26·6%) demonstrated increased markers of coagulopathy, with mild relative hyper-inflammation and dramatically increased markers of end-organ dysfunction (e.g., creatinine, troponin). The odds of 28-day mortality among the class 2 phenotype was more than double that of the class 1 phenotype (40·0% vs.· 23·3%, OR = 2·2, 95% CI [1·2, 3·9]).

**Interpretation:**

We identified distinct phenotypic profiles in COVID-19 associated ARDS, with little variation according to respiratory physiology but with important variation according to systemic and extra-pulmonary markers. Phenotypic identity was highly associated with short-term mortality. The class 2 phenotype exhibited prominent signatures of coagulopathy, suggesting that vascular dysfunction may play an important role in the clinical progression of severe COVID-19-related disease.

**Research in context**

**Evidence before this study**

We searched Google Scholar and PubMed for any prior evidence from 2020 regarding clinical and biological phenotypes in COVID-19-associated ARDS. Search terms included “COVID-19″, “SARS-CoV2”, “ARDS”, “critical illness”, “phenotype”, “immunophenotype”, “heterogeneity”, “inflammation,” “cytokines”, “vascular dysregulation,” “coagulopathy,” and “respiratory dynamics”. No language restrictions were used. We also performed a literature search, without date or language restrictions, on the subject of phenotypes in ARDS. Previous studies established two major biological phenotypes among “classical" ARDS cohorts – hyperinflammatory and hypo-inflammatory – that predict multiorgan failure, mortality, and response to treatment maneuvers. However, little is known about biochemical and clinical phenotypes in COVID-19-associated ARDS.

**Added value of this study**

We identified and characterized biochemical and clinical phenotypes among a large cohort (*n* = 263) of critically ill patients with COVID-19-associated ARDS. We used a multivariate mixture model to identify two phenotypic subgroups based on baseline demographic, respiratory, and laboratory data at ICU admission. We identified a minority phenotype (class 2, 26·6% prevalence) defined by markers of vascular and end-organ dysfunction, with modest relative hyper-inflammation. There was minimal distinction according to respiratory dynamics or ARDS severity. Phenotypic membership was strongly associated with 28-day mortality.

**Implications of all the available evidence**

In this large exploratory analysis, we characterized two distinct phenotypes of COVID-19-associated ARDS, with different clinical and biochemical characteristics despite similar respiratory dynamics, and with markedly different short-term mortality. We outlined a unique phenotypic profile defined by mild hyperinflammation and vascular dysregulation, distinct from established immune phenotypes in classical ARDS. Our findings can aid in early identification of patient subgroups with different clinical outcomes. Future studies are needed to confirm these findings, and to characterize the pathophysiological mechanisms that give rise to the phenotypes that we identified.

Alt-text: Unlabelled box

## 1 Introduction

Substantial biological and pathophysiological heterogeneity exists within the clinical definition of acute respiratory distress syndrome (ARDS) [1,2], limiting targeted treatments. Secondary investigations of landmark ARDS clinical trials defined distinct clinical and biological ARDS phenotypes [3–6]. Identification of ARDS phenotypes based on available laboratory and clinical data could allow for early identification of subpopulations with different clinical outcomes. Furthermore, biological phenotypes provide mechanistic insight to guide targeted therapeutics based on specific pathophysiological processes. ARDS secondary to coronavirus disease-2019 (COVID-19) is similarly characterized by diverse clinical, physiological, and radiographic characteristics [7]. However, the pathophysiology of severe COVID-19 infection is poorly defined.

Among “classical" ARDS cohorts, previous studies have established two predominant biological phenotypes – patients with and without evidence of a hyperinflammatory response – that have retrospectively been correlated with clinical outcomes, including mortality and duration of mechanical ventilation [3] and response to therapeutic strategies, including PEEP [3], fluid management, and statin therapy [8,9]. These two phenotypes were validated in multiple independent datasets [8,5,6], consistently demonstrating 20% higher mortality among the hyperinflammatory subgroup.

Little is known about the phenotypic profile of COVID-19-associated ARDS. It is unclear whether previously established ARDS phenotypes associated with a different degree of inflammation extend to COVID-associated ARDS. Elevated levels of inflammatory cytokines together with other immunologic patterns among COVID-19 patients have been associated with adverse outcomes [10], and debate exists as to whether the cytokine profile associated with COVID-19 ARDS reflects that of non-COVID ARDS [11,12]. Furthermore, in severe COVID-19 disease, substantial thrombotic injury [13] and multi-organ system involvement suggests that vascular dysfunction may be a predominant phenotypic axis [14], [15], [16].

Here, we leveraged a large critical care database to identify and characterize biochemical and clinical phenotypes among critically ill patients with COVID-19-associated ARDS. We used a multivariate mixture model to identify two phenotypic subgroups based on baseline demographic, respiratory, and laboratory data. Our main objective was to determine if we could identify distinct phenotypes of COVID-19 ARDS, consistent with previous studies of both COVID-19 ARDS [7,17] and classical ARDS cohorts [[3], [4], [5],^18^].

## 2 Methods

### 2.1 Data

The study population included patients from the Massachusetts General Hospital (MGH) cohort of the COVID-19 ICU Registry. Briefly, this observational registry reflects treatment and management of COVID-19 patients admitted to the ICU at multiple international centers. Data abstracted from the medical records of ICU patients include demographic factors, daily laboratory markers, physiological parameters, and therapeutic interventions. Data are entered electronically via a StudyTrax (ScienceTRAX LLC, Macon, GA, USA) database created and housed at MGH. Institutional review board approval for data collection was obtained at each site with a waiver of informed consent (Mass General Brigham Protocol 2020P000760). Authors SR, RP, EH, AM, and LB had access to the registry. All data were available from the time of data entry to present.

Demographic, clinical, and physiological data were obtained from patients admitted to any MGH intensive care unit with COVID-19-associated acute respiratory failure between March 13 and August 2, 2020. Patients with a positive SARS-COV2 nasopharyngeal swab at ICU admission, intubation within three days of ICU admission, and a minimum PaO_2_:FiO_2_ < 300 mmHg (*n* = 263), meeting criteria for ARDS, were included in the analyses. Baseline values for variables of interest (Table 1) were defined as the first recorded value for hospital days zero to three. Additional details about the data are provided in the Supplementary Material (Section S1).

**Table 1.** Baseline clinical variables among the study cohort. Mean (standard deviation) reported for normally-distributed continuous variables, with median (IQR) reported for skewed continuous variables (D-dimer, ferritin, fibrinogen, AST, and ALT). Proportions, *N* (%) reported for categorical variables.

Clinical variable	Study cohort, mean (sd) or *n* (%)	*N*
Male gender	175 (66·5%)	263
African American race	33 (12·5%)	263
Hispanic or Latino ethnicity	106 (40·3%)	263
Age (y)	58·8 (15·1)	263
BMI (kg/m^2^)	30·8 (7·39)	263
P_a_O_2_:F_i_O_2_ (mm Hg)	130 (52·9)	263
Driving pressure(mm Hg)	11·3 (3·16)	260
Minute ventilation (L/min)	8·09 (1·96)	263
P_a_CO_2_ (mm Hg)	42·2 (9·18)	263
Ventilatory ratio	1·51 (0·53)	263
Vasopressor requirement	220 (83·7%)	263
pH	7·36 (0·09)	263
Lactate (mmol/L)	1·70 (1·34)	255
Bicarbonate (mmol/L)	23·5 (4·46)	263
WBC (x 10^9^/L)	9·81 (5·76)	263
% Lymphocytes	12·3 (8·58)	260
Hemoglobin (mg/dL)	12·8 (2·20)	263
Platelets (x 10^3^/uL)	228 (111)	263
IL-6 (pg/mL)	118 (159)	53
CRP (mg/dL)	19·6 (35·8)	252
D-dimer (ng/dL)	1460 [925;2883]	263
Ferritin (mg/dL)	965 [478;1657])	246
Fibrinogen (mg/dL)	655 [539;780]	189
LDH (units/L)	660 (1538)	250
PT (s)	15·0 (3·10)	254
aPTT (s)	41·5 (22·3)	223
AST (units/L)*	58·0 [42·2;90·8]	263
ALT (units/L)	40·0 [23·0;62·0]	263
Blirubin (mg/DL)	0·68 (0·62)	263
Albumin (g/dL)	3·21 (0·55)	263
Creatinine (mg/dL)	1·56 (1·74)	263
Hs-Troponin-T (ng/L)	66·1 (219)	243

### 2.2 Statistical analysis

Class-defining variables for latent class identification included baseline demographic features, respiratory parameters, laboratory data, and biomarkers (Table 1). Requirement of continuous intravenous infusion of inotropic-vasopressor medication was also included as a binary variable. Baseline characteristics were reported as mean and standard deviation (continuous variables) or as percentages (categorical variables). Only variables with less than 25% missing data were used for statistical inference. Missing data for class-defining variables were imputed using Multivariate Imputation via Chained Equations [19], and a complete-case analysis was performed as sensitivity analysis (Supplemental Material, Section S4). Interleukin-6 (IL-6) and fibrinogen levels, which were available for *n* = 53 and *n* = 189 patients, respectively, were incorporated post-hoc. Further details on clinical variables and data processing, along with are provided in the Supplemental Material (Section S1). Fig. S2 shows the raw distributions of log-transformed variables and Fig. S3 shows the correlation structure of the class-defining variables.

A two-class multivariate mixture model was used to identify two distinct latent classes based on the variables of interest. A binomial response distribution was used for binary categorical variables (race, ethnicity, gender, and vasopressor requirement) and a Gaussian response distribution was used for continuous variables. Continuous variables were centered and scaled to unit variance prior to model inference. Significantly skewed continuous variables were log-transformed prior to analysis. The model was fit using the expectation-maximization algorithm, initialized at 100 random starting parameter sets to ensure convergence to a global maximum likelihood.

Individuals were allocated to the most likely latent class based on the posterior model probability (probability of class assignment > 50%). Basic comparisons of the raw data between the two identified classes were conducted using the *t*-test or Kruskal-Wallis test for continuous variables, and Fisher's exact test for categorical variables. Two-sided *p*-values < 0·05 were considered statistically significant. The two-class model was compared to a model including an additional latent class based on model selection criteria (Akaike Information Criteria, AIC, and Bayesian Information Criteria BIC), likelihood ratio test, the size of the smallest class, probability of class assignment, and qualitative evaluation of the defining class characteristics. All statistical analyses were conducted in the R statistical programming environment (version 4·0·2).

## 3 Results

Baseline characteristics of the study cohort (*n* = 263) are presented in Table 1. At baseline, oxygenation showed a mean (± standard deviation) P_a_O_2_:F_i_O_2_ equal to 130·0 (± 52·9), and most patients were on intravenous infusion of inotropic-vasopressor agents (83·7%). We identified two latent classes representing 74·4% (Class 1, *n* = 193) and 26·6% (Class 2, *n* = 70) of the cohort, respectively. Posterior probability of class assignment was high (median 98·2%, IQR [98·0%, 100%]), indicating good model fit. We assigned individuals to their most likely latent class based on the posterior probability of class assignment.

**Fig. 1.**
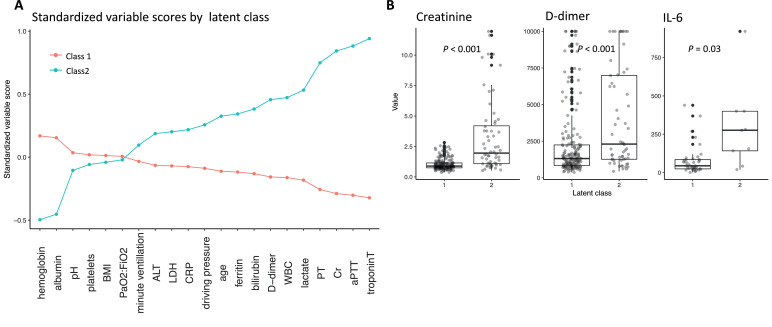
**A:** Differences in the mean standardized values of continuous class-defining variables by latent class. For variable standardization, means are scaled to zero and standard deviations to one. **B:** Distribution of raw data for D-dimer, fibrinogen, and IL-6 by latent class. *P-*values calculated by Kruskall-Wallis test for D-dimer and fibrinogen, and by T-test for IL-6. Boxes denote the inter-quartile range (IQR) of the data, and whiskers extend to 1.5 times the edge of the IQR. Individual outliers are shown as individual points in black. The raw, jittered data are overlain in light gray.

To understand the clinical and biological characteristics that distinguished the two classes, we compared the standardized mean of the continuous class-defining variables (Fig. 1A), and we compared the raw data by assigned class (Table 2). Empirically, Class 2 was distinguished primarily by increased markers of coagulopathy (e.g. D-dimer, median value 2335 ng/L, IQR (1215, 6396) in Class 2 vs 1326 ng/L (864, 2287) in Class 1 (*p* <0·001), PT, mean 17·2, standard deviation 4·89 in Class 2 vs 14·2 s (1·27) in Class 1 (*p* <0·001), and aPTT, mean 57·4, standard deviation 34·0 in Class 2 vs 34·7 s (5·68) in Class 1 (*p* <0·001), Table 2) and end-organ dysfunction (decreased serum pH, mean 7·34, standard deviation 0·09 in Class 2 vs 7·36 (0·08) in Class 1 (*p* = 0·046), Table 2), with increased lactate (mean 2·40 mmol/L, standard deviation 2·31 in Class 2 vs 1·46 mmol/L (0·63) in Class 1 (*p* = 0·001), Table 2), creatinine (mean 3·02 mg/dL, standard deviation 2·82 in Class 2 vs 1·03 mg/dL (0·45) in Class 1 (*p* <0·001), Table 2), and troponin-T (mean 166 ng/L, standard deviation 380 ng/L in Class 2 vs 26·6 ng/L (41·6) in Class 1 (*p =* 0·003), Table 2). There was little distinction according to respiratory parameters, including ARDS severity (P_a_O_2_:F_i_O_2,_ mean 129 mm Hg, standard deviation 63·3 in Class 2 vs 130 mm Hg (48·7) in Class 1 (*p* = 0·900), Table 2). With respect to demographic variables, Class 2 demonstrated a higher prevalence of African American race (20·0% in Class 2 vs. 9·84% in Class 1 (*p* = 0·047), Table 2), and a lower prevalence of Hispanic or Latino ethnicity (24·3% in Class 2 vs. 46·1% in Class 1 (*p* = 0·020), Table 2). There was no significant distinction between classes based on the other categorical class-defining variables (male gender, 74·3% in Class 2 vs. 63·7% in Class 1 (*p* = 0·146), and vasopressor requirement, 90·0% in Class 2 vs. 81·3% in Class 1 (*p* = 0·137), Table 2). Individuals allocated to Class 2 had significantly lower fibrinogen levels compared to Class 1 (median 619, IQR (509, 728) in Class 2 vs. 679 (570, 796) in Class 1 (*p* = 0·014)) and higher IL-6 levels (mean 224 pg/mL, standard deviation 238 ng/L in Class 2 vs 79·3 pg/mL (96·1) in Class 1 (*p =* 0·033), Table 2, Fig. 1B), even though these variables were not used in the initial statistical inference. Next, we evaluated the association between phenotype and clinical outcomes. Allocation to Class 2 was associated with a marked increase in the odds of 28-day mortality (40·0% vs.· 23·3%, OR = 2·2, 95% CI [1·2, 3·9], Supplemental Material Fig. S1).

**Table 2.** Difference in baseline clinical variables between latent subclasses. Class-defining variables are identified in bold. Mean (standard deviation) reported for normally-distributed continuous variables, with median (IQR) reported for skewed continuous variables (D-dimer, ferritin, fibrinogen, AST, and ALT). *P*-values calculated by Student's T-test for normally-distributed continuous variables, by Kruskall-Wallis test for skewed continuous variables, and by Fisher's exact test for categorical variables.

Clinical Variable	Class 1	Class 2	*P* value
	*N* = 193	*N* = 70	
**Male gender**	123 (63·7%)	52 (74·3%)	0·146
**African American race**	19 (9·84%)	14 (20·0%)	0·047
**Hispanic or Latino Ethnicity**	89 (46·1%)	17 (24·3%)	0·020
**Age**	57·0 (15·5)	63·9 (13·0)	0·010
**BMI**	31·0 (7·23)	30·5 (7·84)	0·653
**PaO_2:_ FiO_2_ (mm Hg)**	130 (48·7)	129 (63·3)	0·900
**Driving pressure (mm Hg)**	11·1 (3·01)	11·9 (3·40)	0·060
**Minute ventilation (L/min)**	8·01 (2·03)	8·31 (1·73)	0·233
**P_a_CO_2_ (mm Hg)**	42·6 (8·86)	41·3 (10·0)	0·328
Ventilatory ratio*	1·51 (0·50)	1·50 (0·61)	0·844
**Vasopressor requirement**	157 (81·3%)	63 (90·0%)	0·137
**pH**	7·36 (0·08)	7·34 (0·09)	0·046
**lactate (mmol/L)**	1·46 (0·63)	2·40 (2·31)	0·001
**Bicarbonate (mmol/L)**	23·8 (3·76)	22·5 (4·91)	0·032
**WBC (x 10^9^/L)**	8·75 (4·35)	12·7 (7·84)	<0·001
% Lymphocytes	12·8 (8·29)	10·8 (9·23)	0·113
**Hemoglobin (mg/dL)**	13·2 (1·93)	11·8 (2·53)	<0·001
**Platelets (x 10^3^/*µ*L)**	231 (94·2)	220 (147)	0·563
IL-6 (pg/mL)	79·3 (96·1)	224 (238)	0·033
**CRP (mg/dL)**	16·9 (8·59)	27·3 (66·1)	0·193
**D-dimer (ng/dL)**	1326 [864;2287]	2335 [1215;6396]	<0·001
**Ferritin (mg/L)**	928 [480;1493]	1204 [478;2935]	0·013
Fibrinogen (mg/dL)	679 [570;796]	619 [509;728]	0·014
**LDH (units/L)**	500 (208)	1067 (2840)	0·100
**PT (s)**	14·2 (1·27)	17·2 (4·89)	<0·001
**aPTT (s)**	34·7 (5·68)	57·4 (34·0)	<0·001
AST (units/L)	56·0 [41·0;86·0]	61·5 [44·0;115]	0·130
**ALT (units/L)**	40·0 [24·0;63·0]	40·0 [21·2;61·5]	0·885
**Bilirubin (mg/DL)**	0·62 (0·31)	0·92 (1·19)	0·117
**Albumin (g/dL)**	3·30 (0·45)	2·95 (0·70)	<0·001
**Creatinine (mg/dL)**	1·03 (0·45)	3·02 (2·82)	<0·001
**Hs-Troponin-T (ng/L)**	26·6 (41·6)	166 (380)	0·003

⁎ Derived quantity from P_a_CO_2_ and minute ventilation, not used as an additional class-defining variable in the statistical inference.

**Fig. 2.**
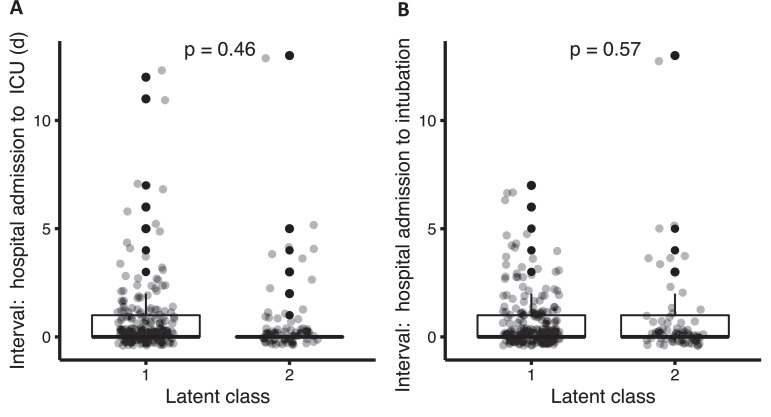
Interval between hospital admission and ICU transfer (**A**) and interval between hospital admission and intubation (**B**) by latent class, with *p-*values calculated by Wilcoxon rank sum test. Boxes denote the inter-quartile range (IQR) of the data, and whiskers extend to 1.5 times the edge of the IQR. Individual outliers are shown as individual points in black. The raw, jittered data are overlain in light gray.

We sought to understand whether the two latent classes represented distinct phenotypic profiles or different stages in disease progression. We therefore compared the time interval between the date of hospital admission and the date of ICU presentation. We found no significant difference between groups (mean 0·74 days in Class 2 vs 0·87 days in class 1, *p* = 0·462, Fig. 2a). Similarly, we found no significant difference in the interval between hospitalization and intubation between the two classes (mean 0·67 days in Class 2 vs 0·86 days in class 1, *p* = 0·572, Fig. 2b). Next, we performed a repeat analysis for class membership later in ICU admission (using class-defining variables recorded at days five to seven, Supplemental Material Section S3, Fig. S5), and found that class switching from class 1 to class 2 occurred in fewer than 10% of cases, indicating phenotypic stability over the initial phase of critical illness.

We also evaluated the best fit for the number of subclasses using latent class analysis (Supplemental Material, Section S2). A three-class model was statistically superior to the two-class model by BIC and likelihood ratio test (Table S2). However, the three-class model produced a small class of only *n* = 17 individuals, and the results were qualitatively similar to the two-class model (Section S2, Fig. S4). A four-class model was not consistently statistically superior to the two-class model (Section S2, Table S1) and resulted in a small class of only *n* = 14. Therefore, we did not find strong evidence in this cohort for additional phenotypes beyond the two described.

We examined differences between the two latent classes in in-hospital interventions (Supplemental Material Section S5). We noted no significant differences in rates of antibiotic use (5·7% in Class 2 vs 7·7% in Class 1, *p* = 0·764), inhaled nitric oxide (NO, 20·0% in Class 2 vs 15·6% in Class 1, *p* = 0·997), or in mean concentrations of heparin infusion (median and standard deviation 252 (149) in Class 2 vs. 184 (107) in Class 1, measured in 100 units/mL, *p =* 0·091) . Continuous renal replacement therapy was more common among the Class 2 phenotype (17·1% in Class 2 vs 0·52% in Class 1, *p* <0·001*)* and corticosteroid use was more common among the Class 1 phenotype (20·0% in Class 2 vs 41·5% in Class 1, *p =* 0·002, Table S2). Of note, for each patient, the first date of steroid administration occurred after the first recorded set of baseline variables, and therefore did not affect phenotypic class assignment. We examined 28-day mortality within each latent class stratified by receipt of corticosteroids and we again found higher mortality associated with the Class 2 phenotype (42.9% in Class 2 vs. 17.5% in Class 1 among patients that received corticosteroids, *p =* 0.032, Supplemental Material Section S5).

## 4 Discussion

We identified two distinct phenotypes of COVID-19-associated ARDS, with substantial differences in biochemical profiles and in short-term mortality, despite minimal differences in respiratory dynamics. The minority phenotype (class 2, *n* = 70, 26·6%) was defined by increased markers of end-organ dysfunction (Fig. 1A, Table 2), relative lactic acidosis, and by increased markers suggestive of coagulopathy (e.g. D-dimer, PT, PTT, fibrinogen). The group of class-defining variables that characterize the class 2 phenotype form a cohesive picture of relative clinical decompensation with altered coagulation, mild relative hyperinflammation, and renal and cardiac impairment. Importantly, phenotypes were associated with short-term mortality. The odds of 28-day mortality among the class 2 phenotype were more than double that of the class 1 phenotype (40·0% vs.· 23·3%, OR = 2·2, 95% CI [1·2, 3·9],).

We found little distinction between phenotypes according to respiratory mechanics (Table 2), or according to the severity of ARDS (P_a_O_2_:F_i_O_2_, Fig. 1A, Table 2). While multiple studies have defined physiological COVID-19 phenotypes according only to respiratory parameters [7,20], our results suggest that meaningful clinical distinctions are also determined by systemic and extrapulmonary processes. This finding is consistent with a recent larger study [21] suggesting that, among classical ARDS cohorts, previously identified biological phenotypes can be extended to critically ill and mechanically ventilated patients that do not meet ARDS criteria.

Our work supports a growing body of evidence that altered coagulation is an important marker of phenotypic variation in COVID-19-associated-ARDS. The more severe class 2 phenotype was distinguished by markedly elevated D-dimer and decreased fibrinogen, as well as elevated PT and aPTT (of note, there was no statistically significant difference in concentrations of heparin infusion, Supplemental Material table S2). In prior literature, increased platelet activation and platelet-monocyte aggregation has been observed in severe COVID-19 infection, but not in mild disease [16]. Other studies have also identified a high burden of thromboembolic disease among postmortem patients with severe COVID-19 infection [22]*.* Furthermore, elevated baseline D-dimer among COVID-19 patients has been shown to predict major coagulation-associated complications, critical illness, and death [23]*.* The Class 2 phenotype was further characterized by relative hypoalbuminemia (Fig. 1A, Table 2), which itself has been associated with hypercoagulability (with marked D-dimer elevation) and higher rates of intensive care treatment and mortality among patients with severe COVID-19 [24]. Taken in context, our results suggest that vascular dysregulation may be a key feature of critical illness and associated morbidity and mortality secondary to COVID-19 infection. Similar coagulation abnormalities have been associated with severity in pre-COVID ARDS and it remains to been seen if these proposed phenotypes may also predict clinical course in non-COVID ARDS [25,26].

We find little role for the traditional hyperinflammatory phenotype established among “classical” ARDS cohorts [3,12,17]. Despite relative hyper-inflammation among Class 2 patients (Table 2, Fig. 1B), IL-6 levels in this cohort were considerably lower than those reported for the hyperinflammatory phenotype in classical ARDS cohorts [27]. This finding is consistent with an exploratory analysis of patients with COVID-19 associated ARDS in the United Kingdom, which found a low prevalence of the traditional hyperinflammatory ARDS phenotype as identified by a parsimonious predictor model validated among classical ARDS cohorts [17]. Similarly, other studies have identified COVID-19-specific inflammatory physiology compared to other hyperinflammatory syndromes, including lower concentrations of circulating inflammatory cytokines and prominent signatures of coagulopathy and dysregulated macrophage activation (with markedly elevated ferritin) [28]. Such differences could explain why IL-6 receptor blockade has had little impact on improving outcomes in patients with severe COVID-19 infection [29]. In contrast to prior analyses of LCA-derived phenotypes among classical ARDS cohorts [3,8], we inferred phenotypes of ARDS arising from a single etiology. Given the departure of our findings from the phenotypes identified in these earlier studies, our findings highlight the importance of controlling for underlying causative triggers in future pathophysiological investigations of ARDS.

The class 2 phenotype was also characterized by significant elevations in serum creatinine and troponin, but no differences in liver injury markers. Whether the renal and myocardial abnormalities are due to microvascular insults in the setting of coagulopathy or more localized organ stress (i.e. hypo/hyper-volemia; persistent tachycardia) is a matter of ongoing investigation [30,31].

Importantly, LCA–derived phenotypes provide an empirical overview of the biological and clinical factors that characterize each latent group. As noted in prior studies [3], while no individual clinical or biological variable is sufficient to classify patients to either latent class, the constellation of variables, considered as a group, form a cohesive cluster that provides a clinical picture of each phenotype. We can conjecture that the variables that differ most between each phenotype (Fig. 2, Table 2) are the ones that can be most reliably used to predict assignment. For example, patients with markedly elevated D-dimer, profound elevations in troponin, or substantially decreased albumin at presentation could raise higher suspicion to the practicing clinician as patients more likely to display the Class 2 phenotype. Future multicohort studies among larger populations will focus on the development of parsimonious predictor models of the Class 1 and Class 2 phenotype, as was seen in extensions of the original derivation of LCA phenotypes among classical ARDS cohorts [8].

Our approach has multiple limitations. First, phenotypes were derived from a single-center cohort. Second, we wish to acknowledge the limitations in terminology regarding our use of the term “phenotype”, and we decided to use this nomenclature given its consistency with previous ARDS literature [3,32]. Particularly, we cannot characterize the Class 2 phenotype formally as a “hypercoagulable” or “vascular” phenotype, but rather our descriptive findings suggest that dysregulation in coagulation is an important phenotypic axis in COVID-19-associated ARDS. Future studies will be needed to better elucidate the nature and pathophysiology of vascular dysregulation. Biomarker data were limited to the variables that had been measured *a priori* in this cohort. We therefore lacked data on more specific biomarkers that could further elucidate differences between inflammatory and vascular responses and we did not assess pulmonary vascular involvement directly via imaging. Similarly, exhaustive clinical data and patient comorbidities, which could be important to distinguish phenotypes, were not available. Vasopressor requirement was available as a binary variable, and therefore Vasoactive-Ionotropic Scores were not compared between subgroups. Laboratory data for each individual were collected according to clinical reasoning in a non-standardized way, which may limit external validity. Phenotypes were evaluated at a single timepoint (baseline, at ICU admission). Therefore, we cannot definitively exclude the possibility that the two latent classes represent different stages in a common disease process, rather than distinct phenotypes. Similarly, data on the time of symptom onset were lacking, which could elucidate differences in the stage of disease progression. However, we found no temporal differences between classes in the interval between hospital admission and ICU admission or intubation, and an analysis of class membership at hospital day 5 showed little evidence of class switching. Future studies could investigate stability of phenotypes over time. It is unclear why a higher rate of corticosteroid use was observed among the Class 1 phenotype (see further discussion in Supplemental Material Section S3). However, in this study, phenotypes were assigned based on baseline data, before the administration of interventions or therapies. Therefore, steroid treatment did not affect phenotypic assignment. Similarly, we examined 28-day mortality within each latent class stratified by receipt of corticosteroids, and we found a higher odds of 28-day mortality associated with the Class 2 phenotype regardless of steroid treatment (Supplemental Material Section S3). Data were missing for some class-defining variables. However, in a sensitivity analysis, the results were robust to exclusion of patients with missing data (for a complete case analysis, see Supplemental Material Section S4 and Fig. S6). Larger studies will be needed to confirm if a three-class model may be a better fit for COVID-19.

In a large cohort of patients with COVID-19 associated ARDS, we identified at the start of ICU admission a minority phenotype defined by increased markers of coagulopathy and end-organ dysfunction, with moderately increased markers of systemic inflammation. Membership in the class 2 phenotype was associated with substantially increased 28-day mortality. Overall, our results suggest that phenotypic profiles in COVID-19 associated ARDS reflect a distinct disease process, with important variation according to systemic and extra-pulmonary markers. Vascular dysfunction may play an important role in severe COVID-19-related disease.

## Funding

This study was supported by the Reginald Jenney Endowment Chair at Harvard Medical School to LB, by LB Sundry Funds at Massachusetts General Hospital, and by laboratory funds of the Anesthesia Center for Critical Care Research of the Department of Anesthesia, Critical Care and Pain Medicine at Massachusetts General Hospital.

## Authors’ contributions

SR, RP, and LB conceived and designed the study. AM, EH, and HD collected, processed, and validated the data. SR performed the statistical inference. All authors contributed to data and results interpretation. SR and LB drafted the manuscript and all authors contributed to revisions and approved the final version.

## Data sharing statement

De-identified individual participant data collected during the trial will be made available upon reasonable request at the time of publication. For access to the data, proposals may be submitted to the corresponding author, Dr. Berra by emailing lberra@mgh.harvard.edu. Data will be made available upon reasonable request to researchers who provide a methodologically sound proposal and whose use of the data has been approved the study authors.

## Declaration of Competing Interest

LB receives salary support from K23 HL128882/NHLBI NIH as principal investigator for his work on hemolysis and nitric oxide. LB receives technologies and devices from iNO Therapeutics LLC, Praxair Inc., Masimo Corp. LB receives grants from “Fast Grants for COVID-19 research” at Mercatus Center of George Mason University and from iNO Therapeutics LLC. BTT reports grants from NIH NHLBI and personal fees from Bayer, Thetis, and Novartis, outside the submitted work. CCH receives research support from AstraZeneca, outside the scope of the submitted work. The other authors have nothing to disclose.

## Appendix Supplementary materials

Image, application 1

## References

[1] Ranieri V.M., Rubenfeld G.D., Thompson B.T. (2012). Acute respiratory distress syndrome: the Berlin definition. JAMA J Am Med Assoc.

[2] Maley J.H., Thompson B.T. (2019). Embracing the heterogeneity of ARDS. Chest.

[3] Calfee C.S., Delucchi K., Parsons P.E., Thompson B.T., Ware L.B., Matthay M.A. (2014). Subphenotypes in acute respiratory distress syndrome: latent class analysis of data from two randomised controlled trials. Lancet Respir Med.

[4] Famous K.R., Delucchi K., Ware L.B. (2017). Acute respiratory distress syndrome subphenotypes respond differently to randomized fluid management strategy. Am J Respir Crit Care Med.

[5] Delucchi K., Famous K.R., Ware L.B., Parsons P.E., Thompson B.T., Calfee C.S. (2018). Stability of ARDS subphenotypes over time in two randomised controlled trials. Thorax.

[6] Bos L.D.J., Scicluna B.P., Ong D.S.Y., Cremer O., Van Der Poll T., Schultz M.J. (2019). Understanding heterogeneity in biologic phenotypes of acute respiratory distress syndrome by leukocyte expression profiles. Am J Respir Crit Care Med.

[7] Gattinoni L., Chiumello D., Caironi P. (2020). COVID-19 pneumonia: different respiratory treatments for different phenotypes?. Intensive Care Med.

[8] Famous K.R., Delucchi K., Ware L.B. (2017). Acute respiratory distress syndrome subphenotypes respond differently to randomized fluid management strategy. Am J Respir Crit Care Med.

[9] Calfee C.S., Delucchi K.L., Sinha P. (2018). Acute respiratory distress syndrome subphenotypes and differential response to simvastatin: secondary analysis of a randomised controlled trial. Lancet Respir Med.

[10] Hue S., Beldi-Ferchiou A., Bendib I. (2020). Uncontrolled innate and impaired adaptive immune responses in patients with COVID-19 acute respiratory distress syndrome. Am J Respir Crit Care Med.

[11] Wilson J.G., Simpson L.J., Ferreira A.M. (2020). Cytokine profile in plasma of severe COVID-19 does not differ from ARDS and sepsis. JCI insight.

[12] Sinha P., Matthay M.A., Calfee C.S. (2020). Is a ‘cytokine Storm’ relevant to COVID-19?. JAMA Intern Med.

[13] Dolhnikoff M., Duarte-Neto A.N., Almeida Monteiro R.A. (2020). Pathological evidence of pulmonary thrombotic phenomena in severe COVID-19. J Thromb Haemost.

[14] Grasselli G., Tonetti T., Protti A. (2020). Pathophysiology of COVID-19-associated acute respiratory distress syndrome: a multicentre prospective observational study. Lancet Respir Med.

[15] Panigada M., Bottino N., Tagliabue P. (2020). Hypercoagulability of COVID-19 patients in intensive care unit: a report of thromboelastography findings and other parameters of hemostasis. J Thromb Haemost.

[16] Hottz E.D., Azevedo-Quintanilha I.G., Palhinha L. (2020). Platelet activation and platelet-monocyte aggregate formation trigger tissue factor expression in patients with severe COVID-19. Blood.

[17] Sinha P., Calfee C.S., Cherian S. (2020). Prevalence of phenotypes of acute respiratory distress syndrome in critically ill patients with COVID-19: a prospective observational study. Lancet Respir Med.

[18] Calfee C.S., Janz D.R., Bernard G.R. (2015). Distinct molecular phenotypes of direct vs indirect ARDS in single-center and multicenter studies. Chest.

[19] Zhang Z. (2016). Multiple imputation with multivariate imputation by chained equation (MICE) package. Ann Transl Med.

[20] Panwar R., Madotto F., Laffey J.G., Van Haren F.M.P. (2020). Compliance phenotypes in early ARDS before the COVID-19 pandemic. Am J Respir Crit Care Med.

[21] Heijnen N.F.L., Hagens L.A., Smit M.R. (2021). Biological subphenotypes of ARDS show prognostic enrichment in mechanically ventilated patients without ARDS. Am J Respir Crit Care Med.

[22] Nadkarni G.N., Lala A., Bagiella E. (2020). Anticoagulation, bleeding, mortality, and pathology in hospitalized patients with COVID-19. J Am Coll Cardiol.

[23] Al-Samkari H., Karp Leaf R.S., Dzik W.H. (2020). COVID-19 and coagulation: bleeding and thrombotic manifestations of SARS-CoV-2 infection. Blood.

[24] Violi F., Ceccarelli G., Cangemi R. (2020). Hypoalbuminemia, coagulopathy, and vascular disease in COVID-19. Circ Res.

[25] Yadav H., Kor D.J. (2015). Platelets in the pathogenesis of acute respiratory distress syndrome. Am J Physiol Cell Mol Physiol.

[26] Ware L.B., Matthay M.A., Parsons P.E., Thompson B.T., Januzzi J.L., Eisner M.D. (2007). Pathogenetic and prognostic significance of altered coagulation and fibrinolysis in acute lung injury/acute respiratory distress syndrome. Crit Care Med.

[27] Sinha P., Delucchi K.L., McAuley D.F., O'Kane C.M., Matthay M.A., Calfee C.S. (2020). Development and validation of parsimonious algorithms to classify acute respiratory distress syndrome phenotypes: a secondary analysis of randomised controlled trials. Lancet Respir Med.

[28] Webb B.J., Peltan I.D., Jensen P. (2020). Clinical criteria for COVID-19-associated hyperinflammatory syndrome: a cohort study. Lancet Rheumatol.

[29] Stone J.H., Frigault M.J., Serling-Boyd N.J. (2020). Efficacy of tocilizumab in patients hospitalized with Covid-19. N Engl J Med.

[30] Ronco C., Reis T., Husain-Syed F. (2020). Management of acute kidney injury in patients with COVID-19. Lancet Respir Med.

[31] Tersalvi G., Vicenzi M., Calabretta D., Biasco L., Pedrazzini G., Winterton D. (2020). Elevated troponin in patients with coronavirus disease 2019: possible mechanisms. J Card Fail.

[32] Prescott H.C., Calfee C.S., Taylor Thompson B., Angus D.C., Liu V.X. (2016). Toward smarter lumping and smarter splitting: rethinking strategies for sepsis and acute respiratory distress syndrome clinical trial design. Am J Respir Crit Care Med.

