# Supplementary material for: Identifying clinical and biochemical phenotypes in acute respiratory distress syndrome secondary to coronavirus disease-2019
Source: eClinicalMedicine. 2021 Apr 15;34:100829. doi: 10.1016/j.eclinm.2021.100829 (PMC8047387; doi:10.1016/j.eclinm.2021.100829)
Supplement: Supplementary file 1 [file mmc1.docx]

**Supplemental Material**

**Figure S1:** Kaplan-Meier curves depicting 28-day survival according to latent class membership. Panel depicts number at risk per latent class.

**Section S1: Additional details regarding data processing and statistical inference**

A total of *n* = 427 patients were admitted to Massachusetts General Hospital intensive care units with respiratory failure secondary to COVID-19 between March 13 and August 2, 2020. Among these, *n* = 282 were intubated within three days of hospital admission and *n* = 263 had a minimum PaO_2_/FiO_2_ < 300 over ICU days 0 – 3, comprising the final cohort that was analyzed. A comprehensive set of baseline clinical variables was chosen to encompass demographic features, respiratory parameters, blood gas, hematologic markers, markers of inflammation, and markers of end-organ function (main text Table 1). With respect to respiratory parameters, ventilation ratio was a derived quantity^1^ calculated as: minute ventilation (ml/min) × PaC_O2_ (mm Hg)]/(predicted body weight (kg) × 100 × 37.5). For the statistical inference, highly correlated/redundant variables were excluded as class-defining variables to mitigate residual confounding. Fig. S2 shows the correlation in the raw data among the full set of baseline variables. For example, baseline AST and ALT were > 95% correlated among the cohort, and therefore ALT was chosen as a representative class-defining variable. Furthermore, variables with ≥ 25% missing data were excluded from the statistical inference to mitigate bias in the imputation of missing values. All excluded baseline variables (ventilatory ratio, IL-6, AST, fibrinogen, % lymphocytes of WBC) were analyzed post-hoc after assignment of individuals to latent subclass. For the statistical inference of latent class, highly skewed or log-scaled continuous variables (D-dimer, ferritin, fibrinogen, AST, and ALT, Fig. S1) were log-transformed, and all continuous variables were transformed to a common z-scale - with mean 0 and standard deviation of one. The multivariate mixture model was fitted to the data using the Expectation Maximization algorithm^2^, an iterative maximum likelihood method for statistical models that depend on unobserved latent variables. To ensure convergence to a global maximum likelihood solution, the algorithm was initiated from 100 different starting parameter sets.


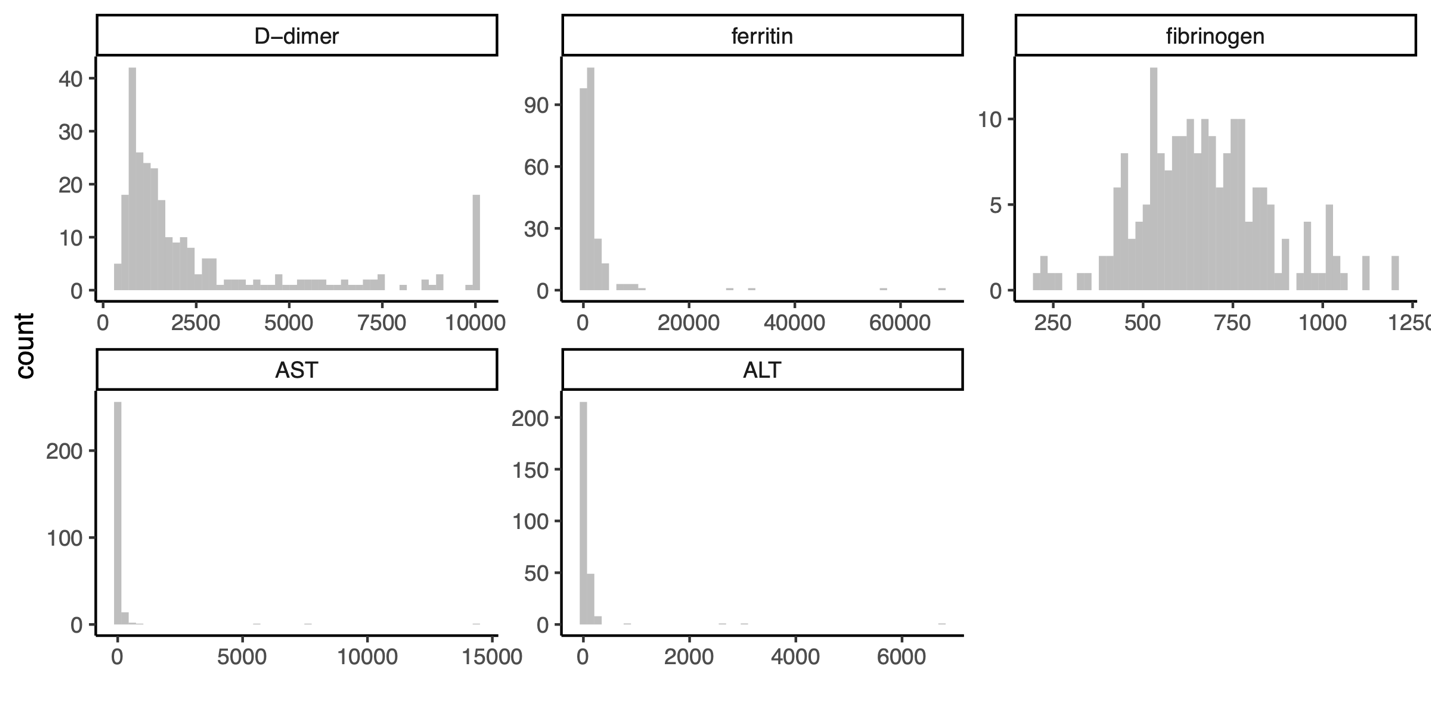


**Figure S2:** Distribution of raw data for skewed and log-distributed continuous variables.

Figure S3: Pairwise correlation between baseline clinical variables (prior to selection of class-defining variables)

**Section S2: Analysis of models with increasing numbers of latent classes**

Consistent with prior literature in both COVID-19-related and classical ARDS^3,4^, our primary objective was to identify two distinct biological phenotypes. However, we also evaluated models with increasing numbers of latent classes. There was a consistent increase in AIC and BIC with increasing numbers of latent classes (Table S1), a phenomenon that has been observed in other studies^4,5^. This effect likely reflects inevitable residual covariation between class-defining variables (for example, covariation between pH, bicarbonate and lactate levels). Variables that portray overlapping approximations of a common underlying condition (e.g. lactic acidosis) are misrepresented by cross-sectional latent class analyses because residual covariation leads the algorithm to identify spurious latent subgroups that actually reflect cut points along a continuous dimension (for example, “less severe” and “more severe” versions of a particular phenotype). In such cases, latent class profiles will differ quantitatively, but not qualitatively, and additional latent classes will not yield truly distinct phenotypes.

Therefore, to choose the most appropriate model, we placed equal weight on the qualitative biological characteristics of each latent class and on the size of the smallest class.

| **Number of latent classes** | **AIC** | **BIC** | ***P-*value** | **Size of smallest class** |
| --- | --- | --- | --- | --- |
| 1 | 17616·78 | 17788·25 |  | 263 |
| 2 | 15412·11 | 15758·61 | < 0·001 | 70 |
| 3 | 14977·78 | 15499·31 | 0·0022 | 13 |
| 4 | 14934·94 | 15631·51 | 0·332 | 9 |

**Table S1:** Statistical comparison of models with varying numbers of latent classes. BIC: Bayesian information criteria. *P*-value calculated by Lo-Mendell-Rubin adjusted likelihood test, comparing the two-class model to a one-class model, and comparing each the three- and four-class models to the two-class model.

Ultimately, the three-class model provided a better statistical fit to the data according to the AIC, BIC and likelihood ratio test. However, the three-class model yielded a smallest class size of only *n* = 13 individuals. Furthermore, the biological profile of the three-class model (Fig. S3) was qualitatively similar to that of the two-class model, demonstrating variation according to markers of coagulopathy (e.g. D-dimer), and end-organ dysfunction (e.g. Cr, troponin T). The minority class (class 2, *n* = 13) showed more extreme variation according to this pattern, but was qualitatively similar to class 1. The four-class model produced a smallest class size of only *n* = 9, and did not provide a statistically significantly better fit to the data than the two-class model (*p* = 0·332, Table S1). Therefore, the two-class model was retained. Furthermore, the high probability of class assignment across individuals in the two-class model (median 98·2%, IQR [98·0%, 100%]) indicated good objective model fit.

**Figure S4:** Differences in the mean standardized values of continuous class-defining variables by latent class for the three-class model. For variable standardization, means are scaled to zero and standard deviations to one.

**Section S3: Analysis of class membership at a secondary timepoint**

The primary analysis investigated phenotypic assignment at baseline ICU admission. To evaluate whether the two identified latent classes represented distinct phenotypes or different stages in clinical disease progression, we re-analyzed class membership at a later point in hospitalization. We used the same multivariate mixture model (main text, Methods) to re-assign phenotypes based on the first value for each class-defining variable recorded between days five and seven. Data were available for *n* = 241 of the original individuals. Of note, two of the original class-defining variables, troponin T and lactate, were missing for > 25% of individuals (for 54·3% and 38·5%, respectively) by day five and were thus excluded from the repeat analysis. Class-specific phenotypic profiles (Fig. S3) were empirically similar to those of the baseline analysis. Among the 241 individuals analyzed in the repeat analysis, we compared phenotypic assignment between the baseline analysis and the repeat analysis. Among 185 individuals originally assigned to Class one, only 18 (9·7%) were re-assigned to Class two at the secondary timepoint. Similarly, among 56 individuals assigned to Class two at the baseline analysis, only 8 (14·2%) were re-assigned to Class one at the secondary timepoint. Therefore, we found relatively little evidence of class-switching over the initial phase of ICU admission.

**Figure S5:** Differences in the mean standardized values of continuous class-defining variables by latent class for the two-class model fitted to data from *n* = 241 patients at the secondary timepoint (first recorded value, hospital days five - seven). For variable standardization, means are scaled to zero and standard deviations to one.

**Section S4: Sensitivity analysis: Complete case analysis**

The model results were robust to a sub-analysis of *n*  = 124 patients with complete data for the class-defining variables. The model identified a minority subclass representing 23·4% (n = 29) of the reduced cohort, consistent with the main analysis. Similarly, the class-specific phenotypic profiles (Fig. S4) were consistent with the main study findings. Therefore, we found no evidence that bias was introduced by the imputation of missing data for variables with < 25% missing values.

**Figure S6:** Differences in the mean standardized values of continuous class-defining variables by latent class for the two-class model fitted to data from only *n* = 124 patients without missing data. For variable standardization, means are scaled to zero and standard deviations to one.

**Section S5: Interventions and therapies by latent class**

|  | **Class 1**  N=193 | **Class 2**  N=70 |  |
| --- | --- | --- | --- |
| Corticosteroid use | 80 (41·5%) | 14 (20·0%) | 0·002 |
| Inhaled NO | 30 (15·6%) | 14 (20·0%) | 0·997 |
| Continuous renal replacement therapy | 1 (0·52%) | 12 (17·1%) | <0·001 |
| Antibiotic use | 15 (7·7%) | 4 (5·7%) | 0·764 |
| Heparin concentration (100 units/mL) | 184 (107) | 252 (149) | 0·091 |

**Table S2**. Difference in clinical interventions between latent subclasses. Mean and standard deviation provided for continuous variables (heparin concentration); *n* and percentage provided for binary categorical variables (corticosteroid use, inhaled NO, continuous renal replacement therapy, antibiotic use). *P*-values calculated by Student’s T-test for continuous variables, and by Fisher’s exact test for categorical variables.

It is unclear why a higher rate of corticosteroid use was observed among the Class 1 phenotype (Table S2). However, for several reasons, it is unlikely that this discrepancy meaningfully affected our conclusions. First, phenotypes were assigned at baseline presentation, before the administration of therapies or interventions. While corticosteroids were typically introduced early in presentation (median time of administration Day 3, interquartile range Day 2-6), for each patient the first date of steroid administration occurred after the first recorded set of baseline variables. Therefore, steroid treatment did not affect phenotypic class assignment. It is possible that steroid administration could have affected the observed mortality differences between classes. To address this point, we examined 28-day mortality within each latent class stratified by receipt of corticosteroids. We again found significantly higher 28-day mortality associated with the Class 2 phenotype. Among patients that did not receive corticosteroid use, the odds ratio of 28-day mortality associated with the Class 2 phenotype compared to the Class 1 phenotype was 1.9 (95% Confidence Interval [1.1, 3.7]). Among patients that did receive corticosteroids, the odds of 28-day mortality was again significantly higher among the Class 2 phenotype (OR = 3.53, 95% CI [1.1, 11.8]), though the variance of the estimate was higher due to limited sample size.

**Supplemental References**

1 Sinha P, Calfee CS, Beitler JR, *et al.* Physiologic analysis and clinical performance of the ventilatory ratio in acute respiratory distress syndrome. *Am J Respir Crit Care Med* 2019; **199**: 333–41.

2 Do CB, Batzoglou S. What is the expectation maximization algorithm? *Nat Biotechnol* 2008; **26**: 897–9.

3 Gattinoni L, Chiumello D, Caironi P, *et al.* COVID-19 pneumonia: different respiratory treatments for different phenotypes? *Intensive Care Med* 2020; **46**: 1099–102.

4 Calfee CS, Delucchi K, Parsons PE, Thompson BT, Ware LB, Matthay MA. Subphenotypes in acute respiratory distress syndrome: Latent class analysis of data from two randomised controlled trials. *Lancet Respir Med* 2014; **2**: 611–20.

5 Hallquist MN, Wright AGC. Mixture Modeling Methods for the Assessment of Normal and Abnormal Personality, Part I: Cross-Sectional Models. *J Pers Assess* 2014; **96**: 256–68.
